# Chilly climate perceived by female engineering undergraduates: an exploratory study using concept mapping

**DOI:** 10.3389/fpsyg.2023.1145795

**Published:** 2023-06-02

**Authors:** Tanhui Kim, Dongil Kim

**Affiliations:** Department of Education, Seoul National University, Seoul, Republic of Korea

**Keywords:** chilly climate, female engineering students, sexism, gender microaggression, concept mapping, South Korea

## Abstract

**Introduction:**

Women still being a minority in engineering majors, they are reported to face discriminatory treatment in a collegiate environment. “Chilly climate,” referring to such a sexist environment, may have a negative impact on women’s mental health, academics, and careers. But, what exactly is it that female students in engineering perceive as chilly, and how chilly is it? This study aimed to explore the chilly campus climate perceived by female undergraduate engineering students in South Korea using the concept mapping method.

**Methods:**

Semi-structured interviews were conducted with 13 participants enrolled for more than four semesters at four-year coeducational universities. After extracting 52 representative statements, the participants were asked to classify them according to content similarity and rate the influence of each statement on their perception of the chilly climate. For concept mapping analysis, multidimensional scaling analysis (ALSCAL), hierarchical cluster analysis (Ward’s method), and non-hierarchical cluster analysis (K-means method) were performed.

**Results:**

Fifty-two statements were extracted under the following four clusters: (i) “Exclusion and alienation inherent in the culture (Cluster 1),” (ii) “Sexual objectification and lack of gender sensitivity (Cluster 2),” (iii) “Male-centered academic situations (Cluster 3),” and (iv) “Prejudice and generalization (Cluster 4).” A concept map was two-dimensional: an X-axis named “context dimension,” with “task: academic” and “non-task: social” at both ends, and a Y-axis named “sexism dimension”, having “explicit” and “implicit” at both ends. The order of higher scores in the influence rating is as follows: Cluster 2, Cluster 3, Cluster 1, and Cluster 4.

**Discussion:**

This study is significant because it conceptualizes the subjective experience of minorities in a collegiate environment and provides influence rating results for prioritized measures. The findings will be helpful in formulating educational policies, psychological counseling, and social advocacy activities. Future research should target larger populations, and cover more diverse cultures, majors, and age groups.

## 1. Introduction

Engineering has traditionally been a male-dominated field, and this has never changed. The percentage of female students enrolled in undergraduate engineering programs has steadily increased from 18.25% (2010) to 22.67% (2018) (National Science Foundation and National Center for Science and Engineering Statistics, 2021), but compared to men, they are still extremely underrepresented. In South Korea, only 21.4% of women enter the engineering department, which is much smaller than that in natural science (50.9%), social and human sciences (57.6%), and medicine (67.7%) (Korea Foundation for Women in Science, Engineering and Technology, 2021). As women are scarce, masculine culture and favoritism toward men are prevalent in the engineering field (Min and Lee, 2005; Duberley and Cohen, 2010; Richman et al., 2011; Hatmaker, 2013; Kim et al., 2016). For example, faculties have lower expectations for female students, provide less academic encouragement or support, and make sexist remarks (Min and Lee, 2005; Park, 2019; Roper, 2019). In Korea, the representative engineering culture includes a military-like hierarchy, obscenity, sexual harassment, and high-intensity drinking (Min and Lee, 2005; Kim et al., 2016; Park, 2019).

“Chilly climate,” a term introduced by Hall and Sandler (1982), refers to receiving such sexist treatment in the collegiate environment. It denotes how traditionally masculine fields are unwelcoming or hostile to women owing to differential treatment by professors or peers in the school environment. Examples of chilly climate include verbal and overt aspects such as disparaging comments about women or sexist jokes and nonverbal and subtle aspects such as paying more attention to men’s comments or waiting longer for men than women to answer a question (Hall and Sandler, 1982). Discriminatory treatment of women exists not only in the classroom but also in various situations such as academic and career counseling, laboratory or fieldwork, group projects, internships, school safety, student autonomy and cultural activities, economic support, and curricula (Hall and Sandler, 1984; Janz and Pyke, 2000). This male-centered atmosphere in schools induces female students to perceive a chilly climate, and has been steadily reported (Goldman, 2012; Cabay et al., 2018; Jensen and Deemer, 2019; Park, 2019; Roper, 2019).

This climate reinforces the marginalization of female engineering students and may have a negative impact on their mental health, academics, and careers. Female students experience negative emotions such as alienation, helplessness, anger, frustration, depression, anxiety, and stress (Hall and Sandler, 1982; Cammaert, 1985; Janz and Pyke, 2000; Walton et al., 2015), and worry about sexism, stereotype threats, and joining the group (O’Brien et al., 2015). Additionally, it causes reduced satisfaction and confidence, deflection, identity confusion, and limited self-expression (Min and Lee, 2005; Chu, 2008; Park, 2019).

Academically, such an environment makes female students doubt their abilities and internalize their devaluation, making them hesitant to participate in academic activities or build relationships with the faculty (Hall and Sandler, 1982; Hall, 2016). Moreover, it has a negative impact on cognitive development (Pascarella et al., 1997), major satisfaction, and self-efficacy (Jeong et al., 2008).

As the chilly environment for women continues to exist in engineering-related workplaces (Makarem and Wang, 2020), it acts as a career barrier for female students (Settles et al., 2006). Female students’ anticipation of career barriers was related to reduced career aspirations and planning (Cardoso and Moreira, 2009; Schuster and Martiny, 2017), and low confidence in future employment, job maintenance, and promotion (Do, 2008). This leads to a “leaky pipeline”; that is, women switch out of Science, Technology, Engineering, and Math (STEM) fields, causing a vicious cycle in which women exist as minorities, leaving fewer female role models to follow (Min and Lee, 2005; Young et al., 2013; Renn, 2014).

Despite the negative effects of the chilly climate on women, men who form the majority negate that sexism is a concern or are indifferent, and some even believe that they have a high sense of gender sensitivity (Hall, 2016; Roper, 2019). This implies that discriminatory words and actions against female students may occur in a subtle or benevolent manner. This phenomenon can be regarded as a “microaggression”; that is, something not particularly intentional but done subtly to make minorities feel uncomfortable (Sue et al., 2007). As microaggression is often perceived as a minor problem, it can lead to feelings of self-doubt, confusion, or alienation (Nadal and Haynes, 2012). In addition, because it is difficult to deal with microaggression promptly or actively, it can be more harmful than overt discrimination (Sue et al., 2007; Dumont et al., 2010). Continuous exposure to microaggression can lead to feelings of low self-esteem, anxiety, and depression (Nadal and Haynes, 2012).

Given that female engineering students experience the negative impact of a chilly climate on their mental health or realization of their potential, it is necessary to thoroughly understand the experiences of these women for more effective psychological intervention and prevention. However, little in-depth research has been conducted on what they perceive as unfavorable to women and the exact circumstances that affect them negatively. Some recent studies on subtle sexism in STEM have focused more on ethnicity or race (Lee et al., 2020; Miles et al., 2020; Marshall et al., 2021), or gender-based microaggression experienced by faculties or professionals (Yang and Carroll, 2018; Makarem and Wang, 2020; Kim and Meister, 2022). In South Korea, research on chilly climates is rare. According to Hwang (2020), 75% of the research on female engineering students deals with career- or academic-related topics, and 75% of the research methods used are quantitative. Allan and Madden (2006) stated that an appropriate research method is crucial when studying a subtle subject, and quantitative methods, such as surveys, may not yield a complete picture.

Therefore, the present study aimed to investigate the chilly climate perceived by female undergraduate engineering students using the concept mapping method, which is useful for identifying the cognitive structure underlying a specific phenomenon, and can present results with minimal researcher subjectivity (Trochim, 1989; Paulson et al., 1999). This study assessed the subjective experience of female engineering students using a qualitative method, processed the data using quantitative statistical methods, and presented the results with a concise pictorial representation. Moreover, the degree of influence of the reported content on the perception of chilly climate was measured to draw more meaningful conclusions.

## 2. Materials and methods

### 2.1. Participants

The participants of this study were female undergraduates enrolled for four semesters or more in the science and engineering department at a 4 year coeducational university. To ensure diversity, students from seven universities, including universities in Seoul, the metropolitan area, and national universities in provincial areas, were selected. A list of science and engineering majors with a female enrollment rate under 30%, based on statistics from the Ministry of Education (2019), was presented. This study was approved by the Institutional Review Board (IRB) of Seoul National University (IRB No. 2012/001-023), and all participants provided written informed consent.

According to Jeong et al. (2008), the longer female students have studied, the more they are influenced by the environment in variables such as major satisfaction and career. Moreover, when research participants were recruited from December 2020 to January 2021, their classes and academic life were shifted to a non-face-to-face manner due to the coronavirus disease 2019 (COVID-19) pandemic. Therefore, students’ experiences with less than four enrollment periods were considered difficult to generalize and were excluded.

Thirteen students from the engineering department were selected as study participants. The number of study participants was determined by referring to Trochim (1989), who stated that there is no strict guideline for concept mapping and 10 to 20 participants are workable. The average age of the participants was 21.4 years old (SD = 1.7 years) and the average semester enrollment period was 5.8 semesters (SD = 1.8 semesters).

### 2.2. Procedure

**Figure 1:**
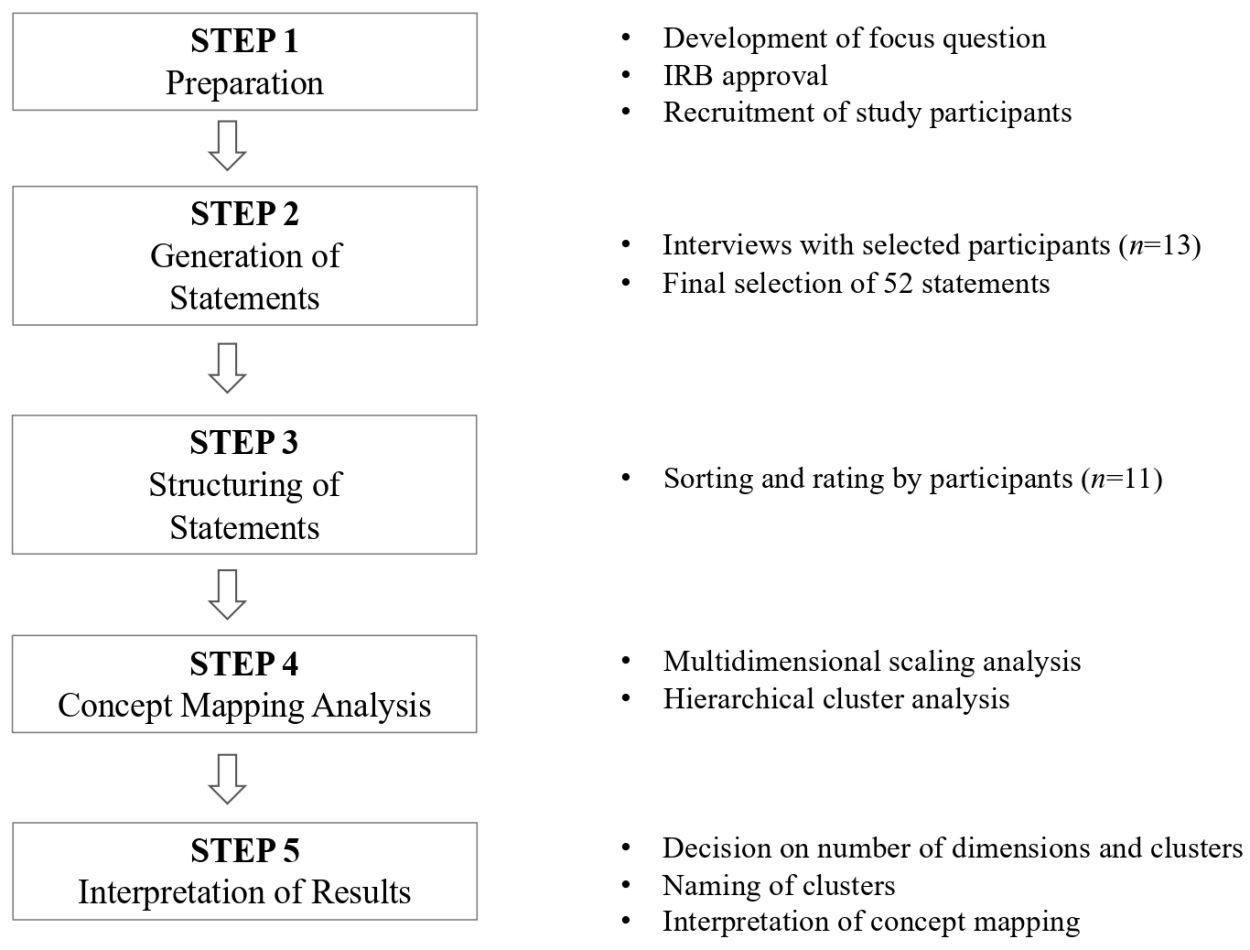
Concept mapping research process.

This study utilized the concept mapping method (Kane and Trochim, 2007) to explore the chilly climate perceived by female undergraduate students in the engineering field. Figure 1 shows the research procedure.

#### 2.2.1. Step 1: preparation

In the preparation stage, a focus question was developed for the interviews. Sample questions were created through literature research, and three experts in qualitative research reviewed the questions. The final focus question selected and used in interviews was as follows: “In what situation (e.g., atmosphere, experience) were you treated differently from men or felt subtle sexism in school as a female undergraduate student in the science and engineering field?”

After obtaining IRB approval, research participants were recruited via recruitment posts on each school’s website.

#### 2.2.2. Step 2: generation of statements

Individual interviews (*n* = 13) were conducted to generate statements. The interviews were conducted between January 5 and 24, 2021. Before the interview, the purpose of the study and interview method were explained in detail through phone calls and notices. If they agreed to participate in the study voluntarily, they were requested to sign the consent form and send it via e-mail before the interview. One day before the interview date, they were sent a focus question via e-mail to allow them to think about the topic in advance.

The interviews were conducted non-face-to-face using Zoom video conference due to COVID-19. After a simple greeting and self-introduction, the participants were allowed to freely talk about the focal question. The researcher tried to promote the production of ideas by asking additional questions related to the topic along with the parts that need to be embodied or clarified. The interview was recorded with the consent of the participants, and took approximately 50–60 min per person.

A total of 183 ideas were extracted from the interviews. According to Kane and Trochim (2007), fewer than 100 statements were appropriate for concept mapping. Thus, the number of statements was abbreviated according to the method of integrating duplicate statements and removing elements mentioned by less than two people (Bedi, 2006). This repeated grouping and reclassifying process was performed by the researcher and two other Ph.D. graduates, and supervised by two professors in the educational counseling department. Fifty-two representative statements were derived using the terms used by the study participants as much as possible.

#### 2.2.3. Step 3: structuring of statements

In this stage, the participants were asked to classify 52 representative statements according to content similarity and rate the influence of each statement on the perception of chilly climate. Owing to personal reasons, only 11 out of 13 participants performed these two tasks from February 25 to March 3, 2021.

Paper cards are generally utilized in similarity classification for concept mapping in offline face-to-face settings. However, this study was conducted when social distancing was recommended due to the COVID-19 pandemic, and there were difficulties in conducting face-to-face interviews with participants from provincial areas. Thus, Microsoft PowerPoint (PPT) was devised as a suitable alternative owing to its familiarity and accessibility to the study participants. Before conducting Step 3, the researcher tested with two participants whether the task instructions were easy to understand and whether tasks using the PPT were easy to perform.

In the similarity classification task, participants were given a PPT file with all 52 representative statements written on the first slide. Each statement was written in a small square card and laid out without overlap so that all statements could be seen at a glance. The participants were then asked to classify these statements by considering a single slide as one group. They were to create as many groups or slides as desired. Referring to Trochim (1989), participants were instructed to place each statement once, and not to place all statements on a single slide or to place one statement on one slide. After classification, participants were asked to name each group.

In the influence rating stage, participants were asked to evaluate on a 7-point Likert scale how much the content of each statement affects their perception of the chilly climate on and off campus (1 = *very little*, 7 = *very much*).

#### 2.2.4. Step 4: concept mapping analysis

For concept mapping analysis, a Similarity Matrix was first created from the classification data. A 52 × 52 binary square matrix was created; 0 was coded for two statements placed in the same group, and 1 was coded for those that did not. A group similarity matrix was produced by summing 13 individual similarity matrices. A multidimensional scale analysis (ALSCAL) on SPSS 22.0 statistical program was used to analyze the number and meaning of the appropriate dimension.

Next, the coordinate values derived from the ALSCAL were used for hierarchical cluster analysis (Ward’s method) and non-hierarchical cluster analysis (K-means method). Ward’s method is suitable for interpreting clusters in conceptual diagrams to classify clusters based on distance (Kane and Trochim, 2007). To secure validity, two-stage clustering was conducted (Hair and Black, 2000). The number of adequate clusters derived from Ward’s method was used to conduct the K-means method.

#### 2.2.5. Step 5: interpretation of results

In Step 5, a concept map was presented on a two-dimensional graph. The group names given by the participants in Step 3 were reflected in naming the clusters. In addition, the impact of each statement and cluster on the perception of a chilly climate was understood from the rating results. Additional explanations are provided in the Results section.

## 3. Results

### 3.1. Multidimensional scaling analysis

In ALSCAL, the stress value is used to determine the appropriate dimensions of the graph. According to Kane and Trochim (2007), the stress value measures the degree to which the distances on the map are discrepant from the values in the input similarity matrix and 0.205–0.376 is an adequate range of the stress value for concept mapping. From the results of ALSCAL analysis, the associated stress value for each dimension was as follows: one dimension, 0.55 (*R*^2^ = 0.38), two dimensions, 0.33 (*R*^2^ = 0.60), three dimensions, 0.22 (*R*^2^ = 0.72), four dimensions, 0.16 (*R*^2^ = 0.79), and five dimensions, 0.13 (*R*^2^ = 0.83). The lowest possible dimensions should be selected because interpretability and simplicity decrease as the number of dimensions increases (Borg and Groenen, 2005). In addition, considering the increase in *R*^2^, the largest increase was observed in two dimensions. Thus, a two-dimensional model was chosen as the most suitable model for this study.

**Figure 2:**
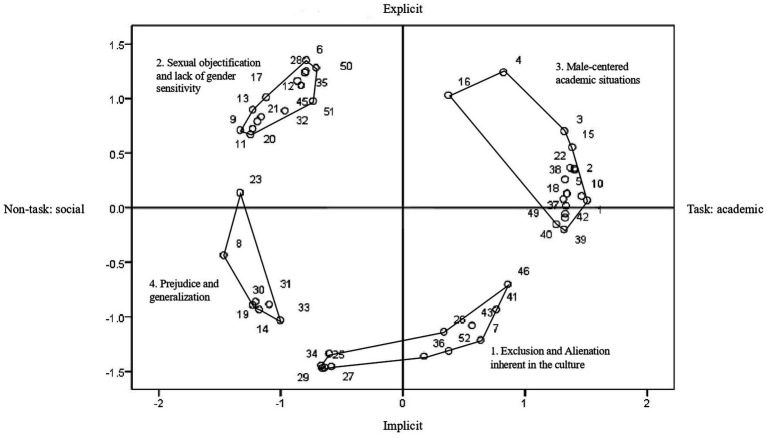
Concept map of the chilly climate perceived by female engineering students.

The researcher examined statements distributed on both the *X*- and *Y*-axes (Figure 2). First, in the positive direction of the *X*-axis, academic situations, such as school classes, team projects, and careers, were prominent. In the negative direction of the *x*-axis, phenomena in non-task contexts, such as interpersonal relationships and perception, were included. Therefore, the *X*-axis was named “context dimension” and considered to represent “task–non-task” contexts on both extremes. For clarity, the subtitles “task: academic” and “non-task: social” were added.

In the case of the *Y*-axis, the positive direction includes more open and direct gender discrimination content found in the words and actions of professors and other male students. Furthermore, the negative direction includes situations such as alienation and a lack of opportunities experienced by female students in the school environment or engineering culture. Therefore, the *Y*-axis was named “sexism dimension,” and “explicit-implicit” sexism was named for both ends.

### 3.2. Hierarchical cluster analysis

As suggested by Yim and Ramdeen (2015), dendrograms and agglomeration schedules were used to determine the appropriate number of clusters. According to the dendrogram presented in SPSS, the number of clusters can be up to six, and according to the scree plot using the coefficient from the agglomeration schedule, the appropriate number of clusters is four. By setting the number of clusters to four, a K-means analysis was performed to form the final cluster.

Clusters were named after scrutinizing the statement contents in each cluster and the attributes of both the *X*- and *Y*-axes. Participants’ feedback on the naming groups was also reflected. Cluster 1 was named “Exclusion and alienation inherent in the culture” (12 statements), Cluster 2 “Sexual objectification and lack of gender sensitivity” (15 statements), Cluster 3 “Male-centered academic situations” (18 statements), and Cluster 4 “Prejudice and generalization” (seven statements). Table 1 presents the statements for each cluster.

**Table 1:** Statements by cluster on perceived chilly climate.

Cluster/statement	M	SD
Cluster 1: Exclusion and alienation inherent in the culture	**5.08**	**0.48**
43. School events in the department consist of male-dominated sports (soccer, basketball, e-sports, etc.).	6.09	1.14
44. To become close, female students should make efforts to meet male students’ interests (game, drinking, sports, etc.).	5.73	1.27
7. Given that female students are a minority, inconveniences in a male-dominated culture or environment are not improved easily.	5.27	1.42
27. Even though female students make male friends, it is difficult to continue the relationship for many reasons (army^a^, etc.).	5.27	1.79
36. There is a strong backlash from male students against programs only for female science and engineering students.	5.27	2.10
29. There is a lack of people with whom female students can openly talk about their experiences and feelings in school.	5.09	1.38
46. The more female students in the group, the more powerfully the female students can express their opinions.	5.00	1.18
25. There is a lack of opportunities for female colleagues to interact.	4.82	1.33
41. There is a lot of news about male seniors who landed nice jobs, but it is hard to hear the same about female seniors.	4.73	1.85
26. Male students’ faults are tolerated more than those of female students.	4.64	1.96
34. Given that there are few female students, it is hard to choose friends.	4.64	1.75
52. Even though there are scholarships for female (engineering) students, the conditions for receipt are strict.	4.45	1.57
Cluster 2: Sexual objectification and lack of gender sensitivity	**5.37**	**0.41**
50. There is an antipathy among male students against feminism.	6.09	0.94
28. Male students rate or rank female students’ appearance.	6.00	0.89
9. Men prefer female students not making their experience of sexual harassment or sexist remarks public.	5.73	1.90
13. Male students exclude and ridicule students who are considered feminists.	5.73	1.35
20. As men lack sensitivity or empathy for gender discrimination and sexual harassment, female students choose to tolerate themselves.	5.55	0.93
24. Female students had experienced or witnessed sexual harassment at a drinking party.	5.45	1.75
21. Even if they are at the scene of sexual harassment or molestation, male students do not interfere or help female students.	5.36	1.96
51. While emphasizing on them being a woman, male students help female students excessively.	5.36	1.36
6. There is little opportunity for open discussion on topics that can cause tension between men and women (e.g., feminism).	5.27	1.68
17. Male students consider and treat female students as potential girlfriends.	5.27	1.27
45. There is a drinking culture where women are split and seated at different tables.	5.18	1.47
35. Male students make comments about appearance to female students freely.	5.09	1.76
32. Male students speak freely about contents or language expressions that may be unpleasant or sensitive to female students.	5.00	1.90
11. Male students think that female students have it easier because they are “women.”	4.73	1.27
12. Male students offer unsolicited help or advice to female students.	4.73	1.27
Cluster 3: Male-centered academic situations	**5.26**	**0.47**
39. There is a small number of or no female professors in the major.	6.09	1.51
3. When a male professor talks about career in class, he speaks mainly to a male audience.	5.82	0.60
5. In the team work of handling machines and tools, the key part is taken by men and the auxiliary part by women.	5.82	0.75
18. Even in the same position or grade level, the opinion of older male students carries more weight than that of female students.	5.82	1.08
1. The position of heads of department or projects are mostly held by male students.	5.64	1.21
2. A professor prefers male students to female students as his graduate students or undergraduate researchers.	5.64	2.06
40. Information and opportunities are centered on male students.	5.64	1.03
16. Although male students insist on doing the hard jobs, they judge female students if they do not participate in the work.	5.18	1.40
48. Female students are generally responsible for auxiliary tasks in a team project.	5.18	1.17
4. A professor makes sexist remarks about ability during class.	5.09	2.26
10. To be recognized on the same level as male students, female students work harder.	5.09	1.38
42. More information and opportunities are provided to male students in career-related school support or consultations with professors.	5.00	1.90
15. The professor uses subjects limited to male students (military, etc.) as an example in the class.	4.91	1.76
49. A group project is naturally led by male students from the beginning.	4.91	1.92
22. As there are only a few female students in the class, the ability or presence of female students is more noticeable to the professor.	4.82	1.54
47. In group project situations, male students are more responsive to men’s opinions than those of women.	4.82	1.99
38. Regarding the lack of female professors in the major, the professor says, “It is because female students do not work hard enough.”	4.64	2.25
37. Feedback on work or idea is provided better to male than female students.	4.55	2.02
Cluster 4: Prejudice and generalization	**4.92**	**0.71**
33. As there is a small number of female students, they are often under the spotlight and easily become the subject of backbiting.	5.82	0.75
31. When a female student is the subject of gossip, false rumors or misunderstandings are more easily generated and spread.	5.73	1.10
30. When a female student talks about the discomfort she feels, male students respond that she is too sensitive.	5.18	1.47
8. Men think women are emotional and cause disturbances (e.g., catfights, and factions).	4.91	1.92
14. Male students consider the words, actions and thoughts of individual female students as those of female (engineering) students as a whole.	4.64	1.36
19. The faults of female students are easily generalized to negative perceptions of women as a whole, even if they are of individuals.	4.27	1.79
23. In private situations such as drinking parties, boys treat girls with extreme caution or do not invite them at all.	3.91	1.14

a Korean men are obligated to go to the army.

### 3.3. Interpretation of concept mapping

Figure 2 presents a concept map of the chilly climate perceived by female undergraduate engineering students. The small circles on the graph indicate the positions of the statements, reflecting the frequency with which participants categorized them into similar groups. Therefore, the closer the positions of the statements, the more similar the participants were considered.

Based on these two dimensions, the statements are distributed in four quadrants. First, the upper-right quadrant corresponds to explicit sexism in the task:academic contexts, which include direct remarks that favor men in academic or career-related situations. Most statements in Cluster 3 (Male-centered academic situations) are included here. Second, the upper-left quadrant shows explicit sexism in non-task:social contexts, such as derogatory remarks against women, sexual harassment, and molestation. The statements of Cluster 2 (Sexual objectification and lack of gender sensitivity) are densely distributed in this quadrant. Third, the lower-left quadrant is for implicit discrimination in non-task:social contexts, including prejudice and generalization of negative perspectives on women. Most statements from Cluster 4 (Prejudice and generalization) and some from Cluster 1 (Exclusion and alienation inherent in the culture) are located here. Fourth, the lower-right quadrant corresponds to implicit discrimination in the task:academic contexts such as marginalization or lack of opportunities for women. Some statements from Cluster 1 (Exclusion and alienation inherent in the culture) and Cluster 3 (Male-centered academic situations) are presented in this quadrant.

### 3.4. Influence ratings results

Table 1 (right-hand side) shows the mean value and standard deviation (SD) of the participants’ rating results for each statement. Except for Statement 23, the average value of every statement was above the median of four on a 7-point Likert scale. Statements 50, 43, and 39 scored the highest average (6.09/7.00).

Cluster 2 (Sexual objectification and lack of gender sensitivity) had the highest mean value M = 5.37, followed by Cluster 3 (Male-centered academic situations) M = 5.26, Cluster 1 (Exclusion and alienation inherent in the culture) M = 5.08, and Cluster 4 (Prejudice and generalization) M = 4.92. Interestingly, the order of the mean value increase and that of the standard deviation decrease were the same. Cluster 2 had the smallest SD value SD = 0.41, followed by Cluster 3 SD = 0.47, Cluster 1 SD = 0.48, and Cluster 4 SD = 0.71.

## 4. Discussion

The purpose of this study was to investigate situations perceived as chilly by female engineering students. To this end, the study was conducted with 13 female students enrolled in engineering majors for over 4 semesters at seven four-year coeducational universities. After one-on-one interviews, 52 statements were extracted. Through the analysis, the study discovered four clusters on a conceptual map. The map was two-dimensional: an *X*-axis named “context dimension,” with “task: academic” and “non-task: social” at both ends, and a *Y*-axis named “sexism dimension,” having “explicit” and “implicit” at both ends. The four clusters, in the order of higher scores in the influence rating, are as follows: (i) “Exclusion and alienation inherent in the culture,” (ii) “Sexual objectification and lack of gender sensitivity,” (iii) “Male-centered academic situations,” and (iv) “Prejudice and generalization.”

First, female engineering students perceived a chilly climate the most by sexual objectification and lack of gender sensitivity. This cluster covers explicit sexism in non-task:social contexts such as blatant hostility toward feminism, sexual harassment or molestation, comments on physical appearance, rude verbal expressions, and being treated as weak or potential girlfriends.

In particular, hostility toward feminism was among the most influential factors in the perception of a chilly climate. Overt antipathy about and ridicule of feminism by male peers and lack of opportunities to openly discuss this matter greatly impacted female students’ perception of the engineering environment as hostile to women. Similarly, in Hall’s (2016) study, men who supported women in STEM were opposed by other men for exhibiting a threat to masculinity. That is, men are considered the dominant gender, and advocating for women is not acceptable in engineering or STEM fields.

In addition, tolerating sexual harassment or sexist incidents rather than making them public had a significant effect on female students’ perception of a chilly climate. This is because most men do not show much support to the victim and prefer not to stir up trouble. Therefore, “self-silencing” is common among women in STEM (Pololi and Jones, 2010), which is not a healthy way to cope with problems.

Moreover, male students evaluating or ranking female students on their looks also had a considerable impact on their perception of the chilly climate. As the remarks are about physical appearance rather than intellect or personality (Hall, 2016) and owing to the assumption that women are okay to be judged, they can be disrespectful and demeaning. In addition, situations such as treating women as potential girlfriends, positioning each woman at a different table at drinking parties, and showing excessive kindness with emphasis on their being women could be considered “subjective objectification” as women are treated as sex objects or possessions (Hall, 2016).

Second, female engineering students perceived a chilly climate in male-centered academic situations, which was found to have the second largest influence. This cluster includes both explicit and implicit sexism in task:academic contexts, and is closest to the definition of chilly climate mentioned by Hall and Sandler (1982). For instance, a professor making remarks about favoring male students in class is explicit sexism in this study and corresponds to the verbal display of chilly climate suggested by Hall and Sandler. In addition, professors’ preference for male students as assistants or the tendency to provide them with more detailed feedback is implicit sexism and falls into the nonverbal aspect of the chilly climate in Hall and Sandler’s study.

This cluster contained the highest number of statements with a high influence on the perception of a chilly climate. Among these, the presence of no or few female professors in the department had the greatest impact. According to previous studies, when providing academic or career-related advice, male professors have a tendency to hold low expectations from female students and encourage them to pursue management instead of technical careers (Kim and Lim, 2011; Cardador, 2017). Female mentors also positively affect undergraduate women in terms of aspiration, achievement (Young et al., 2013), self-concept, grant funding, and promotion (Richman et al., 2011). That is, female professors are not only career role models but also authoritative figures who can understand and help female students. Accordingly, the lack of female professors can further marginalize them.

In addition, in line with previous studies (Hall and Sandler, 1982; Ahn, 2017; Park, 2019), situations such as male students being the leaders most of the time, female students taking auxiliary tasks, information, and opportunities centered on male students were also confirmed in this study and largely affected the perception of a chilly climate.

Third, female engineering undergraduates perceived a chilly climate from “Exclusion and alienation inherent in the culture.” This cluster is related to implicit sexism in various contexts. Examples in the non-task:social context include having difficulties in forming and maintaining relationships with male peers, lack of interaction among female friends, and small options for choosing friends. In the task:academic context, examples include statements about male-centered department events and culture, male students’ opposition to programs only for women, and low availability of these support systems. Previous studies have reported women experiencing hardships in forming a relationship with male colleagues, rare instances of success stories of female graduates, and lack of school support for career development and employment (Han et al., 2010).

In particular, official department events focused only on male students’ participation, which had the greatest impact on female students’ perceptions of the chilly climate. This finding is distinctive and meaningful because it implies that department officials other than male professors or peers can cause a chilly climate. Female students experience a sense of alienation in a male-dominated engineering environment (Hall and Sandler, 1982; Chu, 2008; Park, 2019), and being considered incidental even for official events inevitably makes them feel more marginalized. As a sense of belonging has a direct relationship with feeling how fairly one is treated (Richman et al., 2011), female students strongly perceive a chilly climate. Furthermore, having to adapt to male students’ interests to become close greatly influences female students’ perception of a chilly climate, and limited interaction among female students appeared in several statements.

Fourth, female undergraduates in the engineering field perceived a chilly climate due to prejudice and generalization of women. This cluster includes implicit sexism in non-task:social contexts, such as prejudice about women being emotional and sensitive, rumors about female students, and overgeneralization derived from opinions or faults of a few female students. Prejudice against female students in STEM has been reported in many studies as follows: “Engineering is suitable for men, not women” (Chu, 2008; Blackburn, 2017; Cardador, 2017; Park, 2019), “It should be easier for female students because they are women” (Hall, 2016; Park, 2019), and “Women are not good at math and science” (Walton et al., 2015; Blackburn, 2017; Park, 2019). According to Hall’s (2016) study, women conformed to the social patterns of men to avoid being labeled as “emotional” or a “bitch,” which indicates the prevalence of prejudice and coping skills of women in STEM.

As minorities, being easy targets of gossip and readily created false rumors was found to have a significant effect on the perception of chilly climate. Usually, gossip occurs in informal settings where female students are absent, so there is no opportunity for them to explain or verify the truth. Minorities who are subject to negative stereotypes experience high levels of stress, stereotype threats, and daily adversities (Walton et al., 2015), and female students in STEM experience identity confusion and alienation due to stereotype threats (Johns et al., 2005; Rice et al., 2015; Thackeray, 2016). In other words, female students may experience stress for fear of being or becoming a target, and may be negatively affected due to the fear of confirming prejudice. This is unfair and discriminatory because male engineering students do not necessarily experience the same.

Thus far, the clusters have been mentioned in the order of greatest influence on the perception of chilly climate by female undergraduate students in the engineering field. “Sexual objectification and lack of gender sensitivity” and “Male-centered academic situations,” corresponding to the first and second places, represent explicit sexism, whereas “Exclusion and alienation inherent in the culture” and “Prejudice and generalization,” corresponding to the third and fourth places, are close to implicit sexism. Interestingly, the standard deviation increased in the same order, indicating larger disagreement among the participants. That is, “Sexual objectification and lack of gender sensitivity” had the greatest impact on the perception of chilly climate, and its disagreement degree was small. Conversely, “Prejudice and generalization” had the least influence, and participants’ responses varied greatly. It can be interpreted that, as explicit sexism is delivered more directly, women’s perceptions and influence are not significantly different. However, as implicit sexism is conveyed more subtly, it is not easily recognized (Hall and Sandler, 1982; Swim et al., 2001), resulting in a larger perception variance. Goldman (2012) also states that some undergraduate women in STEM fields are not willing to connect their experience with gender, and if these women do not credit gender for maltreatment or sexism in the field, they experience it without noticing. Though prejudice and generalization scored the last in this study, its contents should not be overlooked as it scored over 4, which is the median value of a 7-point Likert scale. Instead, more attention may be needed as subtle sexism can affect women’s mental health more seriously (Sue et al., 2007; Dumont et al., 2010).

### 4.1. Implications

First, the 52 statements drawn from this study provide useful information and insight into the subjective experience of female undergraduate engineering students. In sum, these students perceived a chilly climate from exclusion and alienation inherent in the engineering culture, sexual objectification and lack of gender sensitivity of their male peers, male-centered academic situations, and prejudice and generalization of women. These findings may shed light on the discriminatory environment and treatment that female engineering students have to endure and draw public attention to make improvements. In addition, they may bridge the perception gap that men and women in the engineering field have and help create climate change from within.

Second, this study is significant because it was the first to explore and expand the understanding of chilly climate using the concept mapping method. This study verified that the perception of chilly climate can be displayed on a two-dimensional diagram, with “task: academic”-“non-task: social” context and “explicit”-“implicit” sexism on both axes. Points on the map indicating the location of each statement and cluster make it easy to understand the degree of similarity of each content. The chilly climate identified in this study encompasses a broader range of sexism than gender microaggression and expands Hall and Sandler’s definition of chilly climate.

Third, scores of influence rating indicate which specific content affects women more in perceiving a chilly climate. The findings may be utilized not only by interested parties in education, but also in psychological counseling. In the counseling scene, the scores can help set the weight and priority of the content needed to identify and understand the client’s problem. They can also be used to develop a scale to facilitate identification of these problems or to develop a counseling program that supports and empowers female engineering students. Furthermore, these findings are expected to be used as evidence for social justice advocacy activities to improve the gender-discriminatory environment faced by female engineering students.

### 4.2. Limitations and future directions

First, as the findings of this study were based on only 13 participants, the results should be interpreted with caution. To cover as diverse experiences as possible, participants were recruited from seven different universities across the country, but this may still be insufficient to reflect the perspectives of a broader population of undergraduate engineering students. In addition, as the participants were recruited based on their voluntary application, and the study relied on self-reports, there may be a bias in reflecting reality.

Second, as this study was conducted with only Korean participants, it may be difficult to generalize the results to all cultures. In a more collectivist culture, the influence of the social environment is stronger (Kim and Choi, 2014). As Korea traditionally has a culture closer to collectivism than individualism, the frustration felt by participants in chilly social relations can be greater. Therefore, the research results should be further tested with larger populations covering more diverse cultures, majors, and age groups.

Third, as this study was conducted during the COVID-19 pandemic, many parts of the study were replaced with non-face-to-face methods. For example, obtaining consent, interviews, similarity classification, and rating processes were all conducted via e-mail, video conference, and other computer programs. These new attempts, different from the traditional concept mapping method, may have affected the participants’ understanding of instructions or concentration. Therefore, efforts to overcome these limitations through repeated research and face-to-face research are required.

## 5. Conclusion

The present study preliminarily examined South Korean female engineering undergraduate students’ perceptions of chilly climate using a concept mapping method and provided a visual representation of the results. Fifty-two final statements from 13 participants were extracted, and their perceptions were grouped into four clusters in a two-dimensional model. The present study conceptualized the subjective experiences of female students in male-dominated campus environments and provided influence rating results so that opportune measures could be prioritized by interested parties in education and mental health care. The unfavorable climate for women can be ameliorated with the help of rigorous future research and social justice advocacy actions.

## Data availability statement

The raw data supporting the conclusions of this article will be made available by the authors, without undue reservation.

## Ethics statement

The studies involving human participants were reviewed and approved by Institutional Review Board of Seoul National University (IRB No. 2012/001-023). The patients/participants provided their written informed consent to participate in this study.

## Author contributions

TK and DK: conceptualization and methodology. TK: data collection, formal analysis, and manuscript writing. DK: supervision. All authors contributed to the article and approved the submitted version.

## Funding

This research was supported by the Ministry of Education of the Republic of Korea and the National Research Foundation of Korea (NRF-2020S1A3A2A02103411).

## Conflict of interest

The authors declare that the research was conducted in the absence of any commercial or financial relationships that could be construed as potential conflicts of interest.

## Publisher’s note

All claims expressed in this article are solely those of the authors and do not necessarily represent those of their affiliated organizations, or those of the publisher, the editors and the reviewers. Any product that may be evaluated in this article, or claim that may be made by its manufacturer, is not guaranteed or endorsed by the publisher.
